# Structural equation modeling of immunotoxicity associated with exposure to perfluorinated alkylates

**DOI:** 10.1186/s12940-015-0032-9

**Published:** 2015-06-05

**Authors:** Ulla B. Mogensen, Philippe Grandjean, Carsten Heilmann, Flemming Nielsen, Pál Weihe, Esben Budtz-Jørgensen

**Affiliations:** Department of Biostatistics, University of Copenhagen, Copenhagen, Denmark; Department of Environmental Health, Harvard School of Public Health, 401 Park Drive, 3E110, Boston, MA 02215 USA; Department of Environmental Medicine, University of Southern Denmark, Odense, Denmark; Pediatric Clinic, Rigshospitalet - National University Hospital, Copenhagen, Denmark; Department of Occupational Medicine and Public Health, Faroese Hospital System, Torshavn, Faroe Islands

## Abstract

**Background:**

Exposure to perfluorinated alkylate substances (PFASs) is associated with immune suppression in animal models, and serum concentrations of specific antibodies against certain childhood vaccines tend to decrease at higher exposures. As such, we investigated the immunotoxic impacts of the three major PFASs in a Faroese birth cohort.

**Methods:**

A total of 464 children contributed blood samples collected at age 7 years. PFAS concentrations and concentrations of antibodies against diphtheria and tetanus were assessed in serum at age 7 years, and results were available from samples collected at age 5. In addition to standard regressions, structural equation models were generated to determine the association between three major PFASs measured at the two points in time and the two antibody concentrations.

**Results:**

Concentrations of all three 7-year PFAS concentrations were individually associated with a decrease in concentrations of antibodies, however, it was not possible to attribute causality to any single PFAS concentration. Hence, the three 7-year concentrations were combined and showed that a 2-fold increase in PFAS was associated with a decrease by 54.4 % (95 % CI: 22.0 %, 73.3 %) in the antibody concentration. If considering both the age-5 and age-7 concentrations of the three major PFASs, the exposure showed a slightly greater loss.

**Conclusions:**

These analyses strengthen the evidence of human PFAS immunotoxicity at current exposure levels and reflect the usefulness of structural equation models to adjust for imprecision in the exposure variables.

**Electronic supplementary material:**

The online version of this article (doi:10.1186/s12940-015-0032-9) contains supplementary material, which is available to authorized users.

## Background

Perfluorinated alkylate substances (PFASs) are applied in water-, soil-, and stain-resistant coatings for clothing and other textiles, oil-resistant coatings for food wrapping materials, and other products. Hence, human PFAS exposures may therefore originate from PFAS-containing products or from environmental dissemination, including house dust, ground water, and seafood [1, 2]. Although systematic toxicity testing has not been carried out, animal models have suggested that immunotoxicity may be an important outcome of PFAS exposures at levels commonly encountered [3]. Pursuant to the above, in the mouse, exposure to perfluorooctane sulfonic acid (PFOS) caused a variety of immunotoxic consequences, including decreased immunoglobulin response to a standard antigen challenge [4, 5]. These associations were reported at serum concentrations similar to, or somewhat higher, than those widely occurring in humans.

In human studies, childhood vaccination responses can be applied as feasible and clinically relevant outcomes, as the children have received the same antigen doses at similar ages [6]. Using this approach, a birth cohort established in the Faroe Islands showed strong negative correlations between serum PFAS concentrations at age 5 years and antibody concentrations before and after booster vaccination at age 5, and 2.5 years later [7]. However, the exposure assessment relied on a single serum sample obtained at age 5. Serial analyses of serum samples from former production workers after retirement suggested elimination half-lives of ~3 years for perfluorooctanoic acid (PFOA) and ~5 years for perfluorooctanesulfonic acid (PFOS) [8], and declines in serum-PFOA concentrations in an exposed community after elimination of the water contamination suggested a median elimination half-life of 2.3 years [9]. Although serum-PFAS concentrations in adults may be fairly stable over time, substantial age-dependent changes occur during childhood [10]. In addition, uncertainty prevails about the relevant exposure window in regard to possible adverse effects in children. Further, binding to serum albumin [11] and body mass index [12] may affect serum concentrations of these substances. Accordingly, imprecision of serum concentrations as exposure indicators must be taken into regard in the data analysis.

Serum-PFAS concentrations of the Faroese birth cohort at age 7 have now been determined, and possible confounders have been ascertained. We can therefore link the immunotoxic outcomes to prospective exposure data. As before [7], we focus on the three major PFASs, i.e., PFOA, PFOS, and perfluorohexanesulfonic acid (PFHxS). Given the fact that three substances were measured postnatally on two occasions and that two different antibody concentrations are available as outcome variables, we complemented standard regression analysis with structural equation models. These models are powerful tools to simultaneously study the associations of several correlated exposures with several outcomes while taking into account exposure uncertainty, missing data, and covariates [13, 14].

## Methods

### Study population

A cohort of 656 children was compiled from births at the National Hospital in Tórshavn in the Faroe Islands during 1997–2000 to explore childhood immune function and the impact on vaccination efficacy [7]. Faroese children receive vaccinations against diphtheria, tetanus, and other major antigens at ages 3 months, 5 months, and 12 months, with a booster at 5 years, as part of the government-supported health care system. All children received the same amount of vaccines and associated alum adjuvant from the same source, although additional vaccines (pertussis and polio) were added to the booster during the project period. Of the 464 children participating in the age-7 examination, 412 had previously undergone the 5-year testing in connection with the booster vaccination. Six children were excluded, as they had more recently received an additional booster vaccination. The study protocol was approved by the Faroese ethical review committee and by the institutional review board at the Harvard School of Public Health; written informed consent was obtained from all mothers.

The PFAS concentrations were measured in serum at age 5 (before the booster vaccination) and age 7. As albumin is likely a major binding protein [11], the serum-albumin concentration was analyzed in all age-7 serum samples to allow adjustment for this possible source of variability. PFAS concentrations were measured by online solid-phase extraction and analysis using high-pressure liquid chromatography with tandem mass spectrometry [7]. Within-batch and between-batch imprecision (assessed by the coefficient of variation) were better than 5.6 % for all analytes. Results with excellent accuracy were obtained in the regular comparisons organized by the German Society of Occupational Medicine.

Serum concentrations of antibodies were measured by the vaccine producer (Statens Serum Institut, Copenhagen, Denmark) using enzyme-linked immunosorbent assay for tetanus and, for diphtheria, a Vero cell-based neutralization assay using 2-fold dilutions of the serum. For both assays, calibration was performed using both international and local standard antitoxins.

### Statistical methods

Antibody concentrations and PFAS exposures were all log-transformed (base 2) before they entered the models. Initial analyses were based on separate multiple linear regressions with an antibody concentration as the dependent variable and a serum-PFAS concentration as a predictor along with age, sex, and booster type. The assumptions of linear dose–response associations were verified by allowing for a more flexible relation between the PFAS and the antibody concentration in generalized additive models using cubic regression splines with three knots [15].

Structural equation models (SEMs) allow for a joint analysis of multiple exposures with several outcome variables [14, 16]. SEMs typically consists of a measurement model in which the observed variables are linked to a limited number of latent variables and a structural model describing the relationship between the latent variables with possible adjustment for the effects of covariates. We considered models with an increasing complexity where the measurement models included an increasing proportion of the available variables from the longitudinal multivariate exposure profile of the subjects.

**Fig. 1:**
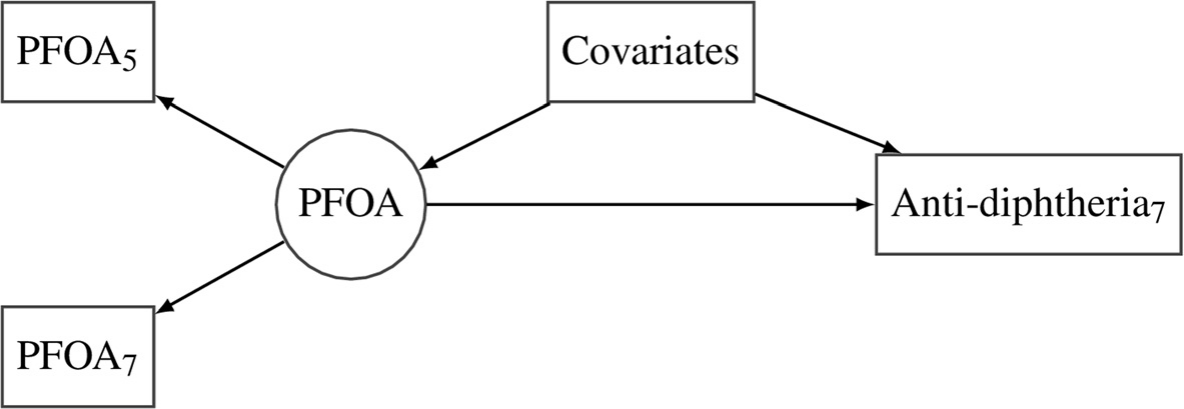
Structural equation model for the association between the latent PFAS concentration and the antibody concentration adjusted for covariates (Additional file 1: Model 1). The model is shown for the relation between latent PFOA (circle) measured by the 5- and 7-year observed concentrations (left squares) and anti-diphtheria (right square). “Covariates” (middle square) are age, sex, and booster type that predicts the latent variable and the two antibody concentrations additively and linearly

Model 1 included both the 5-year and 7-year concentrations of the same PFAS as shown in Fig. 1 for PFOA exposure and its association with the anti-diphtheria antibody concentration (other PFASs and associations with anti-tetanus were modeled similarly). We considered the two observed PFOA concentrations at ages 5 and 7 years: (*log* PFOA_5_, *log* PFOA_7_) as proxy variables for the latent true long-term exposure level (*log*PFOA):

1 $$ log{\mathrm{PFOA}}_5= log\mathrm{PFOA}+{\varepsilon}_5 $$

2 $$ log{\mathrm{PFOA}}_7=\alpha + log\mathrm{PFOA}+{\varepsilon}_7 $$

Thus, after a log-transformation the observed concentration is a sum of the truth and random measurement error. The parameter *α* allows for a general change in the concentration level from age 5 to 7. We further assumed the latent PFOA concentration affect the anti-diphtheria concentration linearly after adjustment for covariates (denoted *C*_*1*_*,…, C*_*k*_*)*:

3 $$ log\ \mathrm{Anti}\hbox{-} {\mathrm{diphtheria}}_7={\beta}_0+{\beta}_1\bullet log\mathrm{PFOA}+{\beta}_2\bullet {C}_1\kern0.5em +\kern0.5em \cdots + {\beta}_{k+1}\bullet {C}_k+\varepsilon . $$

The regression coefficients have the same interpretations as in standard regression analyses, but this model include adjustment for measurement errors, as it is the underlying latent variable, and not the observed exposure which are assumed to affect the outcome. In addition, we allowed for correlations between covariates and exposure variables by assuming that the covariates affected the latent variable (see Additional file 1, p. 3).

The model was extended to allow for the possible dependence for observed PFAS concentrations on the serum-albumin concentration. This was done by modifying the equations (1) and (2) such that the observed PFOA concentrations depended on albumin in addition to the latent PFOA exposure level (see Additional file 1, p. 5). Note that because we modeled the influence of albumin on the observed concentrations, the model allows children with the same underlying exposure to have different measured PFOA concentrations. A similar approach was used for body mass index (body weight divided by the squared height).

To compare the effects of the different PFASs, we combined the individual PFAS models into a single SEM (Additional file 1: Model 2) that allowed antibody concentrations to depend on all three latent PFAS concentrations (see Additional file 1, p. 5–6).

We then considered models where concentrations of the three major PFASs were viewed as reflections of a latent variable representing the overall true PFAS exposure concentration. We first developed a measurement model for 7-year concentrations (*log*PFOA_7_, *log*PFOS_7_, *log*PFHxS_7_)

5 $$ log\ PFO{A}_7={\alpha}_1+{\lambda}_1\bullet log PFAS+{\varepsilon}_1 $$

6 $$ log\ {\mathrm{PFOS}}_7={\alpha}_2+{\lambda}_2\bullet log\mathrm{PFAS}+{\varepsilon}_2 $$

7 $$ log\  PFHx{S}_7={\alpha}_3+{\lambda}_3\bullet log PFAS+{\varepsilon}_3, $$

where *logPFAS* represent the joint latent PFAS concentration and *ε*_1_, *ε*_2_, *ε*_3_ are measurement errors. The latent PFAS variable was then related to the antibody concentrations as in equation (3) (see Additional file 1: Figure S3). This model is similar to the 5-year PFAS exposure model previously used [7].

To investigate if the PFAS exposure at age 5 or 7 years had the strongest effect on antibody concentrations, we included a similar model for the latent 5-year PFAS exposure and allowed antibody concentrations to depend on the latent PFAS variables at both age 5 and age 7; Additional file 1: Model 4 (see Additional file 1, p. 9–10).

**Fig. 2:**
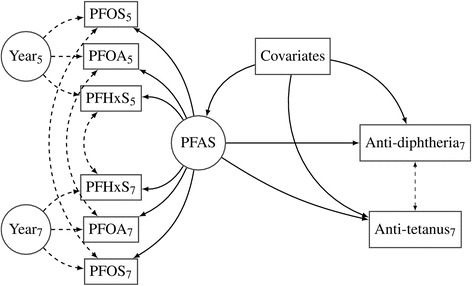
Structural equation model for latent PFAS exposure (middle circle) manifested by concentrations of PFOA, PFOS, and PFHxS at year 5 and 7 (left squares); Additional file 1: Model 5. The dashed (doubled-headed) arrows indicate the local dependencies between the manifest variables. Local dependence between concentrations at the same age is modeled by latent variables Year 5 and Year 7 (left circles). “Covariates” (middle square) are age, sex, and booster type that predicts the joint latent PFAS variable and the two antibody concentrations additively and linearly

As a final model (Additional file 1: Model 5), we extended the joint latent PFAS exposure reflect both sets of serum concentrations at 5 and 7 years, the latter entering the measurement model for the latent PFAS exposure with equations similar to equations 5–7, thus leading to a total of six equations (see full details in Additional file 1, p. 12). We allowed for the possibility that the three concentrations obtained at the same exposure age were correlated (locally dependent) even after conditioning on the latent variable (Fig. 2). This association was assumed to be equally strong for the two exposure assessments. Furthermore, we modeled a local dependence between the age 5 and 7 concentrations for each PFAS. As in the previous models, the latent PFAS variable was then related to the antibody concentrations after adjustment for covariates.

An important advantage of SEMs is the ability to analyze incomplete data through a full-information maximum likelihood (FIML) procedure [16]. We used this option in all the SEMs under the assumption that the data were missing at random [17]. The goodness-of-fit of the models were assessed with the *χ*^2^-test, and the root mean squared error of approximation (RMSEA). The *χ*^2^-test compares the covariance matrix of the observed variables to the covariance matrix predicted from the model. The RMSEA measures the lack-of-fit per degree of freedom. In addition, we report the Bentler Comparative Fit Index (CFI), and the standardized root mean square residuals (SRMR) [16]. Models were considered to have a good fit if the *χ*^2^-test yielded a *p*-value above 5 %, the RMSEA was below 5 %, CFI was above 0.95 and SRMR below 0.08 [16]. All analyses were performed using the statistical software R [18] with the *lava* package [19].

## Results

The characteristics of the children who provided serum for antibody measurements at age 7 years are shown in Table 1. PFOS was by far the most prevalent PFAS with a median serum concentration at age 7 of 15.5 ng/mL (interquartile range (IQR): 12.9, 19.2 ng/mL) which represented a decrease from age 5 (Table 2). Notably, the correlation between the age 5 and age 7 concentrations of the same PFAS was closer than the correlation between the different PFASs at each age.

**Table 1:** Characteristics of children that contributed with a 7-year antibody determination

Variable	Summary
Children (*n* = 459)	
Girls, No. (%)	222 (48.4 %)
Booster vaccine (*n* = 448)	
Booster vaccine type 1, No. (%)	147 (32.8 %)
Serum albumin concentration, median (IQR^a^), g/L	
At age 5 year (*n* = 413)	40.3 (38.8, 42.1)
At age 7 year (*n* = 370)	42.7 (41.2, 44.7)
Antibody concentration at age 7 examination (*n* = 459)	
Age, mean (sd)	7.5 (0.1)
Diphtheria, median (IQR), (IU/mL)	0.8 (0.4,1.6)
Tetanus, median (IQR), (IU/mL)	1.8 (0.6, 4.5)

^a^ *IQR* Inter quartile range

**Table 2:** Pearson correlations for the 5- and 7 year PFAS concentrations. Shown are also the median (IQR) PFAS concentrations

		Pairwise correlation at age 7 year		
Compound	PFAS concentration, ng/mL, median (IQR^a^)	PFOS	PFOA	PFHxS
Age 5 year				
PFOS	17.3 (14.2,21.3)	0.77	0.06	0.43
PFOA	4.1 (3.3, 5.0)	0.34	0.61	0.39
PFHxS	0.6 (5.0, 0.9)	0.40	0.27	0.85
Age 7 year				
PFOS	15.5 (12.8, 19.2)	1		
PFOA	4.4 (3.5, 5.7)	0.29	1	
PFHxS	0.5 (0.4,0.7)	0.50	0.34	1

^a^ *IQR* Inter quartile range

**Fig. 3:**
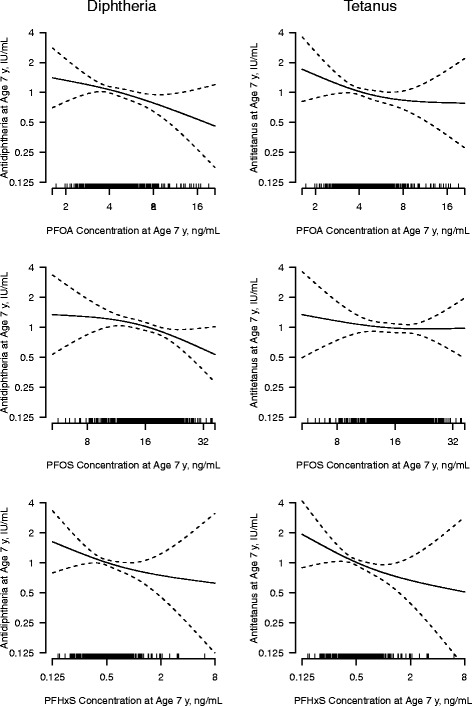
Associations between PFAS and antibody concentrations at age 7 years. Dose–response functions are modeled by generalized additive models with cubic smoothing spline with 3 degrees of freedom, adjusted for age, sex, and booster vaccination type. The dashed lines indicate the 95 % confidence intervals. The spikes on the horizontal line indicate individual observations

Multiple linear regressions showed that higher 7-year PFAS concentrations were associated with lower antibody concentrations. This tendency was most pronounced for anti-diphtheria that decreased by 30.3 % (95 % CI: 7.8 %, 47.3 %), and 25.4 % (95 % CI: 5.8 %, 40.9 %), for a doubling in the PFOS and PFOA concentration, respectively (Table 3). For the anti-tetanus antibody, PFHxS showed the strongest association with a decrease by 22.3 % (95 % CI: 5.2 %, 36.3 %), for a doubling in the exposure. The dose–response relations obtained with generalized additive models verified approximate linearity (Fig. 3).

**Table 3:** The percentage change (% Change) in antibody concentration at age 7 years when the PFAS concentration is doubled. Results are from multiple linear regressions for PFAS concentrations at age 7 years (6 models shown in row 1--2), structural equations models of latent individual PFAS measured by 5- and 7-year concentrations (6 models shown in row 3–4), and one structural equation model with all three latent PFASs (1 model shown in row 5–6). All models were adjusted for the covariates gender, age, and booster type

		PFOS		PFOA		PFHxS	
Antibody	N	% Change	95 % CI	% Change	95 % CI	% Change	95 % CI
Linear regression:							
Anti-diphtheria	443	−30.3	−47.3, −7.8	−25.4	−40.9, −5.8	−16.7	−30.8, 0.2
Anti-tetanus	443	−9.1	−32.8, 23.0	−20.5	−38.2, 2.1	−22.3	−36.3, −5.2
SEM^a^ (Additional file 1: Model 1)							
Anti-diphtheria	448	−34.3	−52.8, −8.9	−34.7	−52.5, −10.2	−13.9	−27.9, 2.7
Anti-tetanus	448	−16.3	−41.5, 19.3	−38.2	−56.1, −13.0	−18.7	−32.8, −1.7
SEM mutually adjusted^b^, Additional file 1: Model 2							
Anti-diphtheria	448	−26.1	−49.4, 7.9	−26.9	−47.4, 1.5	1.7	−17.7, 25.8
Anti-tetanus	448	18.7	−21.2, 78.7	−29.6	−50.6, 0.4	−15.0	−32.4, 6.8

^a^See Fig. 1. Goodness of fits: *χ* ^2^-test *P*: 0.08–0.88, RMSEA: ≤ 0.01–0.06, CFI: 0.99–1.00, SRMR: 0.01–0.02

^b^See Additional file 1: Figure S2. Goodness of fits: *χ* ^2^-test *P*: ≤ 0.01, RMSEA: 0.06, CFI: 0.99, SRMR: 0.03

In the linear regressions we adjusted for gender, age, and booster type. There were significant differences in antibody concentrations between genders where girls had between 23.2 % (95 % CI: 3.8 %, 43.6 %) and 27.2 % (95 % CI: 4.9 %, 44.2 %) lower concentrations. Children that received booster type 1 showed slightly higher antibody concentrations; however, this tendency was only borderline significant for anti-diphtheria.

In structural equation models of individual PFASs (Additional file 1: Model 1; Fig. 1), we focused on the relation of the latent PFAS childhood exposure with the antibody concentrations. Although the serum albumin concentration was positively associated with the observed PFAS measurements, albumin adjustment affected the association of the estimated exposure only to a negligible extent (Table 3 shows the unadjusted estimates, Additional file 1: Table S1 shows the albumin-adjusted estimates). Similarly, we also considered adjustment for BMI, but again with virtually no impact on the results. Consequently, these adjustments were not further considered.

The three latent PFAS variables showed a trend of inverse relationships with each of the antibody concentrations. The effects of PFOS and PFOA were strengthened in comparison to the regression coefficients based on individual 7-year concentrations only. In particular the anti-tetanus concentration showed a decrease of 38.2 % (95 % CI: 13.0 %, 56.1 %), for a doubling in the average childhood PFOA concentration (Table 3, Additional file 1: Model 1). This decrease was nearly twice the magnitude of the decrease obtained in the linear regressions that ignore measurement errors. When the three latent PFASs were mutually adjusted the associations became less apparent and only anti-tetanus antibody showed a borderline significant decrease by 29.6 % (95 % CI: −0.4 %, 50.6 %) at a doubling of PFOA exposure after this adjustment (Table 3, Additional file 1: Model 2). As expected the precision of the coefficients decreased.

As it was not possible to attribute causality to individual PFAS compounds, we combined the data for all three substances at age 7 in a joint latent exposure variable. This total PFAS exposure variable showed the strongest association with anti-diphtheria, which was reduced by more than one half (57.5 %; 95 % CI: 21.2 %, 77.0 %) for a doubling of the PFAS concentration (Table 4, Additional file 1: Model 3). The anti-tetanus concentration similarly showed a decrease by 49.8 % (95 % CI: 2.7 %, 74.1 %), for a doubling in the PFAS concentration.

**Table 4:** Structural equation models for the association between antibodies and latent joint PFAS concentrations. Shown are models for latent PFAS at age 5 years (row 1), latent PFAS at age 7 years (row 2), and latent childhood PFAS (row 3). % Change indicates the percentages change in antibody concentration for a 2-fold PFAS concentration. All models were adjusted for the covariates gender, age, and booster type

		Diphtheria		Tetanus			Joint	
Model	N	% Change	95 % CI	% Change	95 % CI	*P* ^*a*^	% Change	95 % CI
Additional file 1: Model 3								
PFAS at age 5^b^	448	−44.8	−65.8, −11.0	−55.9	−73.6, −26.2	0.40	−50.0	−67.2, 23.8
PFAS at age 7^c^	448	−57.5	−77.1, −21.2	−49.8	−74.1, −2.8	0.64	−54.4	−73.3, −22.0
Additional file 1: Model 5^d^								
PFAS childhood	448	−57.5	−76.9, −21.8	−60.1	−79.3, −22.8	0.64	−55.5	−73.4, −25.5

^a^ *P*-value for the test of same effect on the two antibodies

^b^Goodness-of-fit (same effect model,): *χ* ^2^-test *P*: 0.20, RMSEA: 0.03, CFI: 0.99, SRMR: 0.02

^c^Goodness-of-fit (same effect model): *χ* ^2^-test *P*: 0.41, RMSEA: 0.01, CFI: 0.99, SRMR: 0.02

^d^Goodness-of-fit (same effect model): *χ* ^2^-test *P*: 0.24, RMSEA: 0.02, CFI: 0.99, SRMR: 0.02

The PFAS associations with the two antibody concentrations were similar and could be assumed to be identical (*P* = 0.64). The estimated joint change in antibody concentration then showed a decrease by 54.4 % (95 % CI: 22.0 %, 73.3 %), per doubling of the latent age-7 PFAS exposure.

The impacts of the 5-year PFAS exposure were dramatically reduced when adjusted for the 7-year PFAS exposure (Additional file 1: Model 4, Additional file 1: Table S2). Likewise, the estimated decrease in the antibody concentration per doubling in the 7-year concentration was halved after adjustment of the 5-year concentration. At the same time, the precision of the coefficients decreased, with much wider confidence intervals. As the data did not allow separation of influences attributed to 5-year or concomitant exposure, we combined the 5-year and the 7-year PFAS concentrations into one joint childhood PFAS exposure (Additional file 1: Model 5; Fig. 2). In this model, the adverse impact of PFAS increased, and the precision of the estimates improved. For a doubling of the PFAS exposure, the antibody concentrations decreased by 55.5 % (95 % CI: 25.5 %, 73.4 %).

All analyses were repeated with adjustment also for the pre-booster antibody concentration at age 5 (see Additional file 1, p. 13–15). In this analysis, we modeled the PFAS association with the change in antibody level from age 5 (pre-booster) to age 7 years. For the multiple linear regressions, the precision of all estimates increased, and the regression coefficients of PFOA, and PFOS on anti-tetanus and the regression coefficients of PFOA, and PFHxS on anti-diphtheria were strengthened (Additional file 1: Table S3). For the SEM of latent individual PFASs (Additional file 1: Model 1, Additional file 1: Figure S5), the precision decreased, and estimates were therefore less stable. In the 7-year joint PFAS exposure models (Additional file 1: Model 3), all adverse relations increased while the adverse associations with the joint 5-year PFAS exposure decreased (Additional file 1: Model 3, Additional file 1: Table S4). In the final pre-booster adjusted model (Additional file 1: Model 5), the concentration of antibodies decreased by 51.8 % (95 % CI: 24.6 %, 68.5 %), for a doubling of the joint childhood PFAS exposure, i.e., a slightly weaker association as compared to the unadjusted model.

The SEMs applied generally showed a good fit to the data with *χ*^2^-test *P-*values above 5 % and RMSEA below 5 %. The only exception was the model with mutually adjusted latent PFASs (Additional file 1: Model 4, Additional file 1: Table S4) which did not satisfy the strict *P*-value criterion, but had acceptable values for the other fit-indices.

## Discussion

The focus of the present study was the association between PFAS exposure and the serum-antibody concentration at age 7. The new measurements of concomitant serum-PFAS concentrations at age 7 allowed us to examine the relation to the current PFAS exposures as well as the joint exposures at ages 5 and 7. These analyses showed a stronger inverse association than those obtained with the 5-year PFAS concentration only. However, when the 5-year and the 7-year joint PFAS exposures were mutually adjusted, their substantial correlation made it impossible to distinguish between the influences of each of the two sets of exposure data in regard to the antibody levels. Still, the overall childhood exposures, as reflected by both the age-5 and age-7 PFAS measurements showed the strongest inverse association between PFAS exposure and antibody concentrations.

The amount of antigen in the 5-year booster vaccination was the same for all children. Thus, the 7-year antibody concentrations could be meaningfully adjusted for the 5-year pre-booster antibody level to reveal impacts that could be ascribed to more recent adverse impacts on antibody formation. In this analysis, the joint PFAS association with the anti-tetanus antibody decreased somewhat as compared to the unadjusted results, while the association with the diphtheria antibody remained almost unaffected. This finding suggests that PFASs primarily affect antibody production rather than preceding steps in immune responses to antigen stimulation. In vitro studies suggest that such effects are plausible [20].

Although the impacts of PFAS exposure appear substantial, some residual confounding could still be present. As PFASs in serum are mainly bound to albumin [11], the serum concentration of the former may be affected by the amount present of the latter. However, the association between PFAS exposure and antibody concentrations changed only negligibly after adjustment for the albumin concentration. Similarly, as BMI may also affect the serum concentrations [21], we considered this covariate as a possible confounder. Again, the adjusted results were almost unchanged. All reported results are therefore unadjusted for albumin and BMI.

In addition to the association with the joint PFAS exposure, we explored the individual influences of PFOS, PFOA, and PFHxS. The results did not reveal any clear tendencies, thus suggesting that none of the individual PFASs was the primary explanation of the antibody decrease. Still, PFOA showed a borderline statistically significant association after adjustment for the other PFASs. Most experimental studies focused on PFOS and PFOA [3] and very little are known about the immunotoxicity of PFHxS. The mechanisms of action are unclear. While PFOS and PFOA in humans may only in part act via the same pathways [20], it is unknown if PFHxS shares mechanisms of action with the other PFASs. Thus, whether a joint analysis of PFAS exposures has a toxicological foundation is also uncertain. Nonetheless, the fact that the combined exposure parameter in the SEMs showed a stronger association with the antibody concentrations would seem to support some degree of additive effects. Further, the standard regression analyses suggest that each individual PFAS has immunotoxic impacts of its own.

Structural equation models proved to be very useful in the analysis of these data with multiple exposures measured at different time points and a multivariate outcome. A main advantage of this framework is that information from many variables can be combined into a joint analysis which potentially becomes more powerful than standard regression modeling. In addition, SEMs allow for measurement error in covariates, which is essential for unbiased effect estimation, especially in models that include correlated independent variables [22]. We considered a number of different models to describe the association between the longitudinal multivariate exposure profile and the antibody response at age 7 years. These analyses indicated that the causative dose is a long-term average where all three PFASs may contribute. Such an analysis would not be meaningful in standard regression models, as it would require that all exposure variables were included simultaneously as independent variables. The present study illustrates how to develop SEMs where observed exposures were assumed to be correlated with underlying causative latent variables. Some models grouped variables according to chemical type (PFOS, PFOA or PFHxS) while others combined information from the same year of exposure (5 or 7 years). We believe that all the models are relevant as they show different aspects of the relationship between PFAS exposure level and antibody concentration. The model selection process resulted in a model where all exposure variables were related to one causative latent variable. However, this does not mean that we consider the final model to be superior in general. Thus, had one of the underlying analyses clearly indicated that a single chemical concentration or only a single exposure age was important, it would not have been appropriate to develop a model combining all variables.

The increased power of SEMs comes from the fact that all collected variables are exploited but also from additional assumptions necessary for inclusion of all variables simultaneously. According to a recent critique, SEMs often make unrealistic assumptions about the relationships between the covariates [23]. This concern is not relevant to our analysis, which allowed a completely flexible model for the covariates. However, multiple exposures were included by postulating the existence of one or more sets of underlying latent variables. Although a reasonable assumption, the existence of such unobserved variables is difficult to prove, but statistical fit indices indicated that these models provided a good approximation to the data distribution. Our findings therefore suggest that carefully conducted SEM analyses with a minimal number of weak assumptions provide an important supplement to standard regression results, especially when involving imprecise assessment of multiple exposures.

In support of our findings of PFAS immunotoxicity, a recent study of 99 Norwegian children at age 3 years found that the maternal serum PFOA concentrations were associated with decreased vaccine responses in the children, especially toward rubella vaccine, as well as increased frequencies of common cold and gastroenteritis [24]. However, PFOS and PFOA concentrations in serum from 1400 pregnant women from the Danish National Birth Cohort were not associated with the total hospitalization rate for a variety of infectious diseases in 363 of the children up to an average age of 8 years [25]. In adults, PFOA exposure was associated with lower serum concentrations of total IgA, IgE (females only), though not total IgG [26]. Also, elevated serum-PFOA serum concentrations were associated with a reduced antibody titer rise after influenza vaccination [27]. Thus, overall, support is building that PFAS exposure may be associated with deficient immune functions, although the clinical implications need to be defined in detail.

Our study is limited to PFAS exposure assessments at two points during childhood at an interval of about 30 months. While taking into regard the elimination half-lives of the PFASs of 2–5 years [8, 9], these two measurements may not fully characterize the childhood exposure profile that is most relevant to humoral immunity functions. The addition of the age-7 serum concentrations clearly improved the association between PFAS exposure and the antibodies, thus suggesting that the imprecision of the exposure estimate had decreased. As exposures during childhood are likely to vary [1, 10], it is possible that serial serum-PFAS analyses would provide even stronger evidence for PFAS immunotoxicity. However, distinguishing between exposures at different ages and between the associations attributed to different PFASs will require greater variability and lower correlation between the individual exposure parameters.

The 5-year booster vaccination is in principle the last booster vaccination that a Faroese child receives, and long-term protection is therefore anticipated. Our previous results [7] showed that many children at age 7 had antibody concentrations below the level assumed to provide the desired protection. Thus, while the exact magnitude of the serum-antibody concentration may not be clinically important, very low levels will mean poor or absent protection. Our findings show that PFAS exposure may inhibit the formation of antibodies and cause more children to be unprotected despite a full regimen of vaccinations. While tetanus and diphtheria may not be a serious hazard in the Faroese and many other countries, the strongly decreased antibody concentrations reflect a severe immunological deficit, one that is much stronger than the one associated with PCB exposure [28]. As optimal immune system function is crucial for health; the associations identified should be regarded as adverse. We recently calculated benchmark dose levels to estimate the magnitude of exposure limits that would protect against the immunotoxicity observed [29]. The results suggested that current exposure limits may be more than 100-fold too high. The improved adjustment for imprecision of the exposure assessment in the present study adds support to the notion that substantially strengthened prevention of PFAS exposures is indicated.

## Conclusions

The vulnerable window of time for the immunotoxic effects of PFASs is unknown, and exposure assessment must therefore take into account temporal changes in serum concentrations. Structural equation models provided a useful approach to statistical analysis as they take into account that each PFAS measurement is likely to an imprecise indicator of the causative exposure. These models also allow for a multivariate response, and incorporate incomplete data. By incorporation of serum concentrations at two points in time, the results confirmed and extended findings in standard regression models where antibody concentrations decreased as a function of PFAS concentrations. This tendency was strengthened in the structural equation models that included both sets of serum concentrations. These analyses add further evidence to the notion that immunotoxicity may occur in humans at the current exposure levels.

## Additional file

Additional file 1:
**Structural equation modeling of immunotoxicity with exposure to perflourinated alkylates.**
