# Supplementary material for: Structural equation modeling of immunotoxicity associated with exposure to perfluorinated alkylates
Source: Environ Health. 2015 Jun 5;14:47. doi: 10.1186/s12940-015-0032-9 (PMC4488050; doi:10.1186/s12940-015-0032-9)
Supplement: Additional file 1: — Structural equation modeling of immunotoxicity with exposure to perflourinated alkylates. [file 12940_2015_32_MOESM1_ESM.pdf]

Supplemental Material

Structural equation modeling of immunotoxicity with exposure to perflourinated alkylates

Ulla B. Mogensen, Philippe Grandjean, Carsten Heilmann,
Flemming Nielsen, Pal Weihe, Esben Budtz-Jørgensen

Contents

Model 1: Individual latent PFAS concentrations **3**

Equations of Model 1 PFOA 3

Adjustment for serum albumin in Model 1 4

Figure S1: path diagram of albumin adjusted Model 1 4

Equations of albumin adjusted Model 1 PFOA 5

Table S1: results from albumin adjusted Model 1 5

Model 2: Mutually adjusted individual latent PFASs **6**

Figure S2: path diagram of Model 2 6

Equations of Model 2 7

Model 3: Joint PFAS concentration age 7 years **8**

Figure S3: path diagram of Model 3 8

Equations of Model 3 8

Model 4: Mutually adjusted joint-year PFAS concentrations **9**

Figure S4: path diagram of model 4 9

Equations of Model 4 10

Table S2: results of Model 4 11

Model 5: Joint childhood PFAS concentration **12**

Figure S5: path diagram of model 5 12

Equations of Model 5 13

Adjustment for the pre-booster anti-body concentrations **14**

Table S3: results of Model 1–2 after pre-booster adjustment 15

Table S4: results of Model 3–5 after pre-booster adjustment 16

Model 1: Individual latent PFAS concentrations

In the main article we refer to “Model 1 PFOA”, “Model 1 PFOS”, and “Model 1 PFHxS” for the three equivalent model 1’s respectively. Equations are only shown for PFOA association to anti-diphtheria as example. The path diagram for Model 1 PFOA is shown in the main article in Figure 1.

Equations of Model 1

$$\log \text{PFOA}_5 = \log \text{PFOA} + \varepsilon_5 \tag{1}$$

$$\log \text{PFOA}_7 = \alpha + \log \text{PFOA} + \varepsilon_7 \tag{2}$$

$$\log \text{Anti-diphtheria}_7 = \beta_0 + \beta_1 \cdot \log \text{PFOA} + \beta_2 \cdot \text{C}_1 + \cdots + \beta_{k+1} \cdot \text{C}_k + \varepsilon_d \tag{3}$$

$$\log\ \text{PFOA} = \zeta_0 + \zeta_1 \cdot \text{C}_1 + \cdots + \zeta_k \cdot \text{C}_k + \varepsilon_l \tag{4}$$

The $\varepsilon_5, \varepsilon_7, \varepsilon_d, \varepsilon_l$ are normally distributed random errors. Furthermore, $C_1, \ldots, C_k$ denote the covariates.

Adjustment for serum albumin in Model 1

Figure S1: path diagram of albumin adjusted Model 1

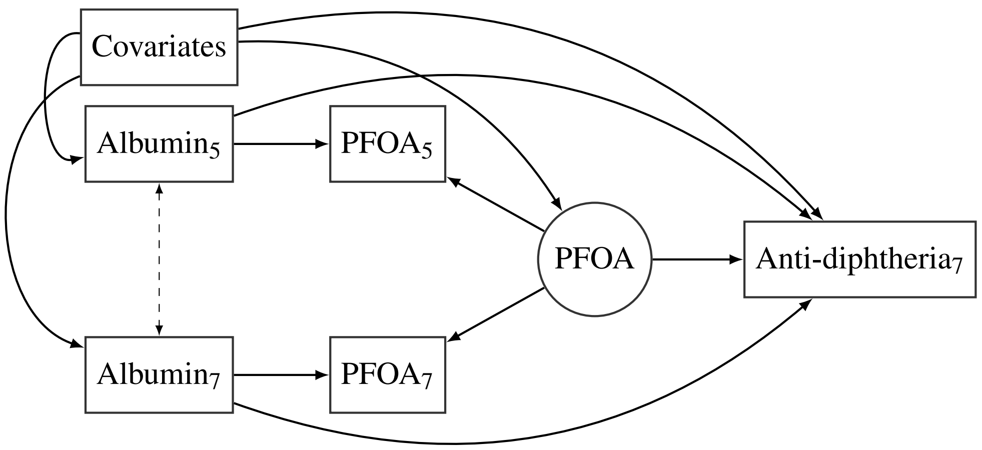

Figure S1: Structural equation model for association between PFAS exposure and antibody concentration adjusted for albumin level. The model is shown for the relation between latent PFOA (circle) measured by the 5- and 7-year observed concentrations (middle squares). The observed PFOA concentrations are adjusted for observed albumin concentrations (left squares). "Covariates" (upper left square) are age, sex, and booster type that predicts albumin and the latent PFOA concentrations additively and linearly.

Equations of albumin adjusted Model 1

$$\log \text{PFOA}_5 = \log \text{PFOA} + \alpha_5 \cdot \log \text{Albumin}_5 + \varepsilon_5 \tag{5}$$

$$\log \text{PFOA}_7 = \alpha_0 + \log \text{PFOA} + \alpha_7 \cdot \log \text{Albumin}_7 + \varepsilon_7 \tag{6}$$

$$\begin{aligned}\log \text{Anti-diphtheria}_7 = {} & \beta_0 + \beta_1 \cdot \log \text{PFOA} \\ & + \beta_2 \cdot \log \text{Albumin}_5 + \beta_3 \cdot \log \text{Albumin}_7 \\ & + \beta_4 \cdot \text{C}_1 + \cdots + \beta_{k+3} \cdot \text{C}_k + \varepsilon_d \end{aligned} \tag{7}$$

$$\log\ \text{PFOA} = \zeta_0 + \zeta_1 \cdot \text{C}_1 + \cdots + \zeta_k \cdot \text{C}_k + \varepsilon_l \tag{8}$$

$$\log \text{Albumin}_5 = \zeta_{0,5} + \zeta_{1,5} \cdot \text{C}_1 + \cdots + \zeta_{k,5} \cdot \text{C}_k + \varepsilon_{c5} \tag{9}$$

$$\log \text{Albumin}_7 = \zeta_{0,7} + \zeta_{1,7} \cdot \text{C}_1 + \cdots + \zeta_{k,7} \cdot \text{C}_k + \varepsilon_{c7} \tag{10}$$

The $\varepsilon_5, \varepsilon_7, \varepsilon_d, \varepsilon_l, \varepsilon_{c5}, \varepsilon_{c7}$ are normally distributed random errors where we estimate $Cov(\varepsilon_{c5}, \varepsilon_{c7}) = \tilde{\Omega}$. Furthermore, $C_1, \ldots, C_k$ denote the covariates.

Table S1: results from albumin adjusted Model 1

Table S1: Results from the three Model 1s adjusted for serum albumin. Change is the change in percentages in antibody concentration when the latent PFAS concentration is doubled.

		**PFOS**		**PFOA**		**PFHxS**	
**Antibody**	**N**	**% Change**	**95% CI**	**% Change**	**95% CI**	**% Change**	**95% CI**
Anti-diphtheria	448	−34.3	−52.5, −9.0	−34.8	−52.8, −10.1	−13.9	−27.9, 2.9
Anti-tetanus	448	−14.8	−40.2, 21.2	−36.5	−55.1, −10.2	−17.6	−31.9, −0.3

Goodness-of-fit: χ^2-test $P: 0.15 - 0.96$ RMSEA: $\leq 0.001 - 0.04$, CFI: 0.99 - 1.00, SRMR: 0.01 - 0.02

Model 2: Mutually adjusted latent individual PFASs

Figure S2: path diagram of Model 2

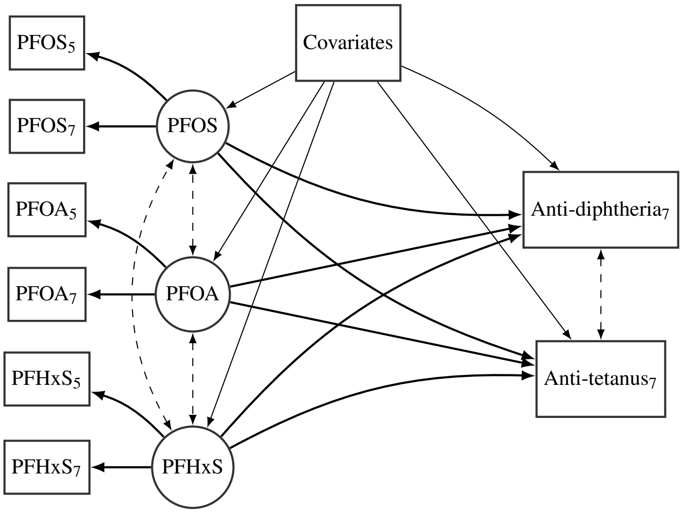

Figure S2: Structural equation model with three latent variables (circle), PFOA, PFOS, and PFHxS, each measured by the respective 5- and 7-year concentrations (left squares). Subscripts indicate the year of the measurements. "Covariates" (middle square) are age, sex, and booster type that predicts the latent variables and the two anti-body concentrations additively and linearly.

Equations of Model 2

$$\log \text{PFOA}_5 = \log \text{PFOA} + \varepsilon_{1,5} \tag{11}$$

$$\log \text{PFOA}_7 = \alpha_1 + \log \text{PFOA} + \varepsilon_{1,7} \tag{12}$$

$$\log \text{PFOS}_5 = \log \text{PFOS} + \varepsilon_{2,5} \tag{13}$$

$$\log \text{PFOS}_7 = \alpha_2 + \log \text{PFOS} + \varepsilon_{2,7} \tag{14}$$

$$\log \text{PFHxS}_5 = \log \text{PFHxS} + \varepsilon_{3,5} \tag{15}$$

$$\log \text{PFHxS}_7 = \alpha_3 + \log \text{PFHxS} + \varepsilon_{3,7} \tag{16}$$

$$\begin{aligned} \log \text{Anti-diphtheria}_7 &= \beta_0 + \beta_1 \cdot \log \text{PFOA} + \beta_2 \cdot \log \text{PFOS} + \beta_3 \cdot \log \text{PFHxS} \\ &\quad + \beta_4 \cdot \text{C}_1 + \cdots + \beta_{k+3} \cdot \text{C}_k + \varepsilon_d \end{aligned} \tag{17}$$

$$\begin{aligned} \log \text{Anti-tetanus}_7 &= \gamma_0 + \gamma_1 \cdot \log \text{PFOA} + \gamma_2 \cdot \log \text{PFOS} \gamma_3 \cdot \log \text{PFHxS} \\ &\quad + \gamma_4 \cdot \text{C} + \cdots + \gamma_{k+3} \cdot \text{C}_k + \varepsilon_t \end{aligned} \tag{18}$$

$$\log\ \text{PFOA} = \zeta_{0,1} + \zeta_{1,1} \cdot \text{C}_1 + \cdots + \zeta_{k,1} \cdot \text{C}_k + \varepsilon_{l,1} \tag{19}$$

$$\log\ \text{PFOS} = \zeta_{0,2} + \zeta_{1,2} \cdot \text{C}_1 + \cdots + \zeta_{k,2} \cdot \text{C}_k + \varepsilon_{l,2} \tag{20}$$

$$\log\ \text{PFHxS} = \zeta_{0,3} + \zeta_{1,3} \cdot \text{C}_1 + \cdots + \zeta_{k,3} \cdot \text{C}_k + \varepsilon_{l,3} \tag{21}$$

The $\varepsilon_5, \varepsilon_7, \varepsilon_d, \varepsilon_t, \varepsilon_{l,1}, \varepsilon_{l,2}, \varepsilon_{l,3}$ are normally distributed random errors where we estimate $Cov(\varepsilon_d, \varepsilon_t) = \Omega_1$, and $Cov(\varepsilon_{li}, \varepsilon_{lj}) = \sigma_{ij}, i, j = 1, 2, 3$. Furthermore, $C_1, \ldots, C_k$ denote the covariates.

Model 3: Joint PFAS concentration age 7 years

Figure S3: path diagram of Model 3

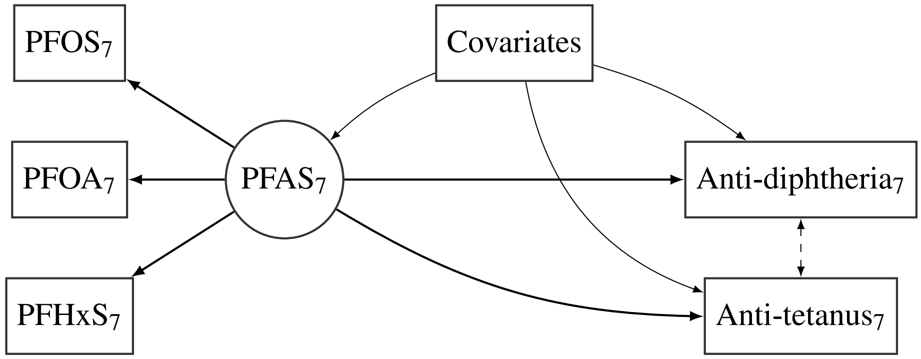

Figure S3: Structural equation model with a latent 7 year PFAS exposure (circle) manifested by the three observed 7 year concentrations PFOA, PFOS, and PFHxS (left squares). "Covariates" (middle square) are age, sex, and booster type that predicts the latent variable and the anti-body concentrations additively and linearly.

Equations of Model 3

$$\log \text{PFOA}_7 = \alpha_1 + \lambda_1 \cdot \log \text{PFAS}_7 + \varepsilon_1 \quad (22)$$

$$\log \text{PFOS}_7 = \alpha_2 + \lambda_2 \cdot \log \text{PFAS}_7 + \varepsilon_2 \quad (23)$$

$$\log \text{PFHxS}_7 = \alpha_3 + \lambda_3 \cdot \log \text{PFAS}_7 + \varepsilon_3 \quad (24)$$

$$\log \text{Anti-diphtheria}_7 = \beta_0 + \beta_1 \cdot \log \text{PFAS}_y + \beta_2 \cdot \text{C}_1 + \cdots + \beta_{k+1} \cdot \text{C}_k + \varepsilon_d \quad (25)$$

$$\log \text{Anti-tetanus}_7 = \gamma_0 + \gamma_1 \cdot \log \text{PFAS}_7 + \gamma_2 \cdot \text{C} + \cdots + \gamma_{k+1} \cdot \text{C}_k + \varepsilon_t \quad (26)$$

$$\log\ \text{PFAS}_7 = \zeta_0 + \zeta_1 \cdot \text{C}_1 + \cdots \zeta_k \cdot \text{C}_k + \varepsilon_l \quad (27)$$

The $\varepsilon_1, \varepsilon_2, \varepsilon_3, \varepsilon_d, \varepsilon_t, \varepsilon_l$ are normally distributed random errors where we estimate $Cov(\varepsilon_d, \varepsilon_t) = \Omega$. Furthermore, $C_1, \ldots, C_k$ denote the covariates.

Model 4: Mutually adjusted joint-year PFAS concentrations

Figure S4: path diagram of model 4

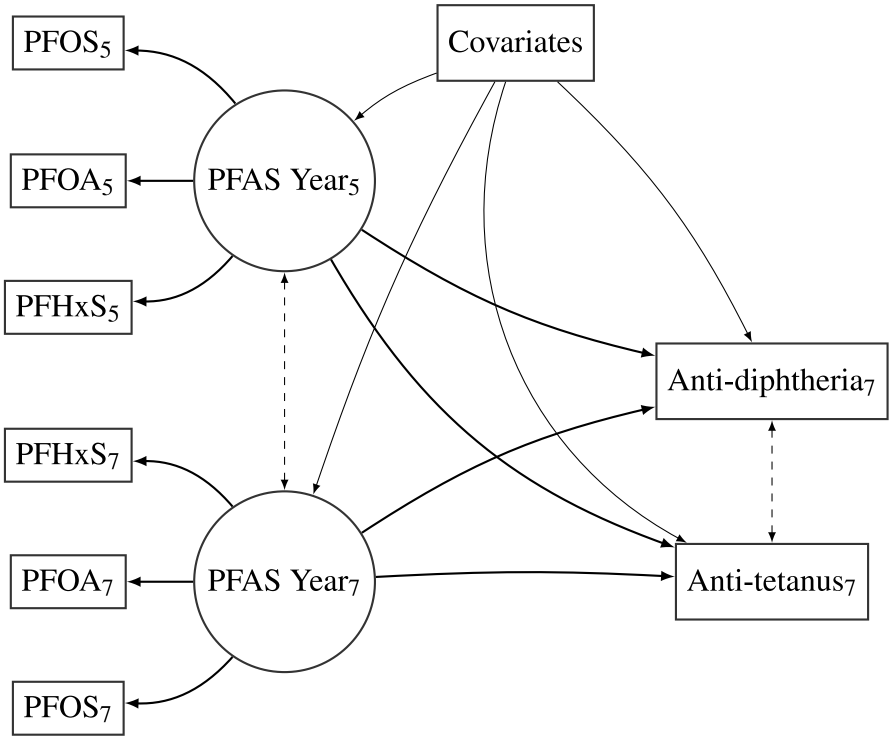

Figure S4: Structural equation model including a joint latent PFAS concentration at age 5 years (circle; Year 5), and a joint latent PFAS concentration at age 7 years (circle; Year 7). The two latent PFAS concentrations are manifested by the observed PFOA, PFOS, and PFHxS concentrations at age 5 years, and at age 7 years respectively. "Covariates" (middle square) are age, sex, and booster type that predicts the latent variables and the two anti-body concentrations additively and linearly.

Equations of Model 4

$$\log \mathrm{PFOA}_5 = \alpha_{5,1} + \lambda_{5,1} \cdot \log \mathrm{PFAS}_5 + \varepsilon_{5,1} \tag{28}$$

$$\log \mathrm{PFOS}_5 = \alpha_{5,2} + \lambda_{5,2} \cdot \log \mathrm{PFAS}_5 + \varepsilon_{5,2} \tag{29}$$

$$\log \mathrm{PFHxS}_5 = \alpha_{5,3} + \lambda_{5,3} \cdot \log \mathrm{PFAS}_5 + \varepsilon_{5,3} \tag{30}$$

$$\log \mathrm{PFOA}_7 = \alpha_{7,1} + \lambda_{7,1} \cdot \log \mathrm{PFAS}_7 + \varepsilon_{7,1} \tag{31}$$

$$\log \mathrm{PFOS}_7 = \alpha_{7,2} + \lambda_{7,2} \cdot \log \mathrm{PFAS}_7 + \varepsilon_{7,2} \tag{32}$$

$$\log \mathrm{PFHxS}_7 = \alpha_{7,3} + \lambda_{7,3} \cdot \log \mathrm{PFAS}_7 + \varepsilon_{7,3} \tag{33}$$

$$\begin{aligned} \log \text{Anti-diphtheria}_7 &= \beta_0 + \beta_1 \cdot \log \mathrm{PFAS}_5 + \beta_2 \cdot \log \mathrm{PFAS}_7 \\ &\quad + \beta_3 \cdot \mathrm{C}_1 + \cdots + \beta_{k+2} \cdot \mathrm{C}_k + \varepsilon_d \end{aligned} \tag{34}$$

$$\begin{aligned} \log \text{Anti-tetanus}_7 &= \gamma_0 + \gamma_1 \cdot \log \mathrm{PFAS}_5 + \gamma_2 \cdot \log \mathrm{PFAS}_7 \\ &\quad + \gamma_3 \cdot \mathrm{C} + \cdots + \gamma_{k+2} \cdot \mathrm{C}_k + \varepsilon_t \end{aligned} \tag{35}$$

$$\log\ \mathrm{PFAS}_5 = \zeta_{0,5} + \zeta_{1,5} \cdot \mathrm{C}_1 + \cdots + \zeta_{k,5} \cdot \mathrm{C}_k + \varepsilon_5 \tag{36}$$

$$\log\ \mathrm{PFAS}_7 = \zeta_{0,7} + \zeta_{1,7} \cdot \mathrm{C}_1 + \cdots + \zeta_{k,7} \cdot \mathrm{C}_k + \varepsilon_7 \tag{37}$$

$$\tag{38}$$

The $\varepsilon_{5,1}, \cdots, \varepsilon_{5,5}, \varepsilon_{7,1}, \ldots, \varepsilon_{7,3}, \varepsilon_d, \varepsilon_t, \varepsilon_5, \varepsilon_7$, are normally distributed random errors where we estimate $Cov(\varepsilon_d, \varepsilon_t) = \Omega$. Furthermore, $C_1, \ldots, C_k$ denotes the covariates.

Table S2: results of Model 4

Table S2: Results of structural equation model (Model 4) where antibodies concentrations depend on a latent joint 5 year PFAS concentrations (Year 5) and on a latent joint 7-year PFAS concentration (Year 7). Change indicates the percentages change in antibody concentration for a 2-fold PFAS concentration.

		Diphtheria		Tetanus		*P* value for	Joint	
**Model 4**	**N**	**% Change**	**95% CI**	**% Change**	**95% CI**	**same effect**	**% Change**	**95% CI**
PFAS at age 5	448	0.2	−48.6, 95.1	−40.5	−71.0, 22.0	0.13	−6.2	−49.0, 72.5
PFAS at age 7	448	−58.8	−83.9, 5.4	−15.7	−68.6, 126.4	0.13	−55.8	−81.9, 7.7

[a] Goodness-of-fit (same effect model) $p :< 0.01$ (χ^2-test), RMSEA: 0.05, CFI: 0.99, SRMR: 0.03.

Model 5: Joint childhood PFAS concentration

Figure S5: path diagram of model 5

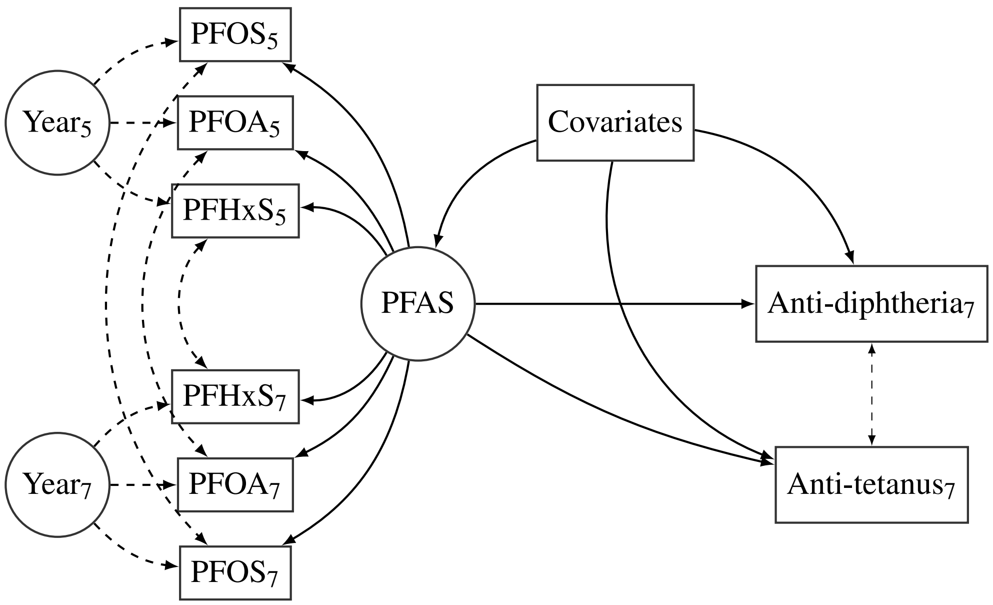

Figure S5: Structural equation model of a joint latent PFAS concentration (middle circle) manifested by the observed PFOA, PFOS, and PFHxS concentrations at age 5 years (upper left squares), and at age 7 years (bottom left squares) respectively. The dashed (doubled-headed) arrows indicate the local dependencies between the manifest variables. Local dependence between concentrations at the same age is modeled by latent variables Year$_5$ and Year$_7$ (left circles). "Covariates" (middle square) are age, sex, and booster type that predicts the joint latent PFAS variable and the two anti-body concentrations additively and linearly.

Equations of Model 5

$$\log \text{PFOA}_5 = \alpha_{5,1} + \lambda_{5,1} \cdot \log \text{PFAS} + \delta_{5,1} \cdot \text{Year}_5 + \varepsilon_{5,1} \tag{39}$$

$$\log \text{PFOS}_5 = \alpha_{5,2} + \lambda_{5,2} \cdot \log \text{PFAS} + \delta_{5,2} \cdot \text{Year}_5 + \varepsilon_{5,2} \tag{40}$$

$$\log \text{PFHxS}_5 = \alpha_{5,3} + \lambda_{5,3} \cdot \log \text{PFAS} + \delta_{5,3} \cdot \text{Year}_5 + \varepsilon_{5,3} \tag{41}$$

$$\log \text{PFOA}_7 = \alpha_{7,1} + \lambda_{7,1} \cdot \log \text{PFAS} + \delta_{7,1} \cdot \text{Year}_7 + \varepsilon_{7,1} \tag{42}$$

$$\log \text{PFOS}_7 = \alpha_{7,2} + \lambda_{7,2} \cdot \log \text{PFAS} + \delta_{7,2} \cdot \text{Year}_7 + \varepsilon_{7,2} \tag{43}$$

$$\log \text{PFHxS}_7 = \alpha_{7,3} + \lambda_{7,3} \cdot \log \text{PFAS} + \delta_{7,2} \cdot \text{Year}_7 + \varepsilon_{7,3} \tag{44}$$

$$\log \text{Anti-diphtheria}_7 = \beta_0 + \beta_1 \cdot \log \text{PFAS} + \beta_2 \cdot \text{C}_1 + \cdots + \beta_{k+1} \cdot \text{C}_k + \varepsilon_d \tag{45}$$

$$\log \text{Anti-tetanus}_7 = \gamma_0 + \gamma_1 \cdot \log \text{PFAS} + \gamma_2 \cdot \text{C}_1 + \cdots + \gamma_{k+1} \cdot \text{C}_k + \varepsilon_t \tag{46}$$

$$\log\ \text{PFAS} = \zeta_0 + \zeta_1 \cdot \text{C}_1 + \cdots + \zeta_k \cdot \text{C}_k + \varepsilon_l \tag{47}$$

The $\varepsilon_{5,1}, \cdots, \varepsilon_{5,5}, \varepsilon_{7,1}, \ldots, \varepsilon_{7,3}, \varepsilon_d, \varepsilon_t, \varepsilon_l$, are normally distributed random errors where we estimate $Cov(\varepsilon_d, \varepsilon_t) = \Omega$ and $Cov(\varepsilon_{5,i}, \varepsilon_{7,i}) = \sigma_i, i = 1,2,3$. Furthermore, $C_1, \ldots, C_k$ denotes the covariates.

Adjustment for the pre-booster anti-body concentrations

All models presented in the article and this supplemental material are repeated with an adjustment of the pre-booster (age 5 year) anti-body concentrations. All models are adjusted for the pre-booster concentration by including the 5-year anti-body concentration as an additional predictor of the 7-year anti-body concentrations. For example for the linear regression analysis of PFOA:

$$\begin{aligned}\log \text{Anti-diphtheria}_7 = \beta_0 + \beta_1 \cdot \log \text{PFOA} + \beta_3 \cdot \log \text{Anti-diphtheria}_5 \\ + \beta_3 \cdot \text{C}_1 + \cdots + \beta_k \cdot \text{C}_{k-3} + \varepsilon \end{aligned} \tag{48}$$

where ε is the residual term.

Results are shown for all models in supplemental material, Table S3 and S4.

Table S3: results of Model 1–2 after pre-booster adjustment

Table S3: The percentage change (% Change) in 7-year antibody concentration when the individual PFAS concentration is doubled. The results are obtained from multiple linear regressions (6 models shown in row 1-2), structural equations models of latent individual PFAS measured by 5- and 7-year concentrations (6 models shown in row 3-4), and one structural equation model with all three latent PFASs (1 model shown in row 5.6). The 7-year antibody concentration is adjusted for the 5-year pre-booster antibody concentration in all the analyses.

		PFOS		PFOA		PFHxS	
**Anti-body**	**N**	**% Change**	**95% CI**	**% Change**	**95% CI**	**% Change**	**95% CI**
**Pre-booster adjusted multiple linear regression**							
Anti-diphtheria	398	−28.9	−44.8; −8.4	−26.5	−41.2; −8.2	−24.1	−35.6; −10.4
Anti-tetanus	398	−11.0	−30.3; 13.8	−24.0	−38.7; −5.8	−21.6	−33.2; −8.1
**Pre-booster adjusted Model 1** [a]							
Anti-diphtheria	403	−30.1	−48.0, −6.0	−33.9	−51.3, −10.4	−21.4	−32.8, −8.1
Anti-tetanus	403	−13.6	−35.1, 15.0	−36.0	−52.3, −14.1	−18.3	−29.7, −5.0
**Pre-booster adjusted Model 2** [b]							
Anti-diphtheria	401	−12.7	−28.7, 6.8	−13.7	−39.1, 22.4	−21.6	−43.2, 8.0
Anti-tetanus	401	−15.2	−30.1, 2.9	18.8	−15.1, 66.2	−27.6	−46.8, −1.4

[a] Three models: see supplemental material, Figure S5. Goodness-of-fit: χ^2-test $P: 0.08-0.72$, RMSEA: $\leq 0.01-0.05$, CFI: 0.99 – 1.00, SRMR: 0.01 - 0.2

[b] One model: see supplemental material Figure S2, and S5. Goodness-of-fit: χ^2-test $P :< 0.01$, RMSEA: 0.05, CFI: 0.99, SRMR: 0.03

Table S4: results of Model 3–5 after pre-booster adjustment

Table S4: Structural equation models for the association between antibodies and latent joint PFAS concentrations adjusted for the 5 year pre-booster anti-body concentrations. Shown are models for latent PFAS at age 5 (row 1), latent PFAS at age 7 (row 2), and latent childhood PFAS (row 3). Change indicates the percentages change in antibody concentration for a 2-fold PFAS concentration.

		**Diphtheria**		**Tetanus**		***P* value for**	**Joint**	
**Model**	**N**	**% Change**	**95% CI**	**% Change**	**95% CI**	**same effect**	**% Change**	**95% CI**
**Pre-booster adjusted Model 4**[a]								
PFAS at age 5	401	23.2	−30.2, 117.6	−20.3	−53.9, 37.7	0.25	−1.9	−38.4, 56.1
PFAS at age 7	401	−68.6	−86.3, −28.1	−34.7	−69.7, 40.4	0.25	−53.8	−76.3, −9.8
**Pre-booster adjusted Model 5**								
PFAS at age 5 [b]	401	−39.1	−58.9, −9.7	−39.2	−58.4, −11.3	0.98	−39.2	−56.0, −16.0
PFAS at age 7 [c]	401	−63.6	−79.6, −35.1	−53.9	−73.2, −20.5	0.43	−58.8	−74.4, −33.7
**Pre-booster adjusted Model 6**[d]								
PFAS childhood [d]	401	−55.3	−73.4, −25.0	−48.0	−68.1, −15.3	0.63	−51.8	−68.4, −26.4

[a] See supplemental material, Figure S4, and S5. Goodness-of-fit (same effect model) $p: 0.03$ (χ^2-test), RMSEA: 0.03, CFI: 0.99, SRMR: 0.03.

[b] Similar to supplemental material, Figure S3. Goodness-of-fit (same effect model): χ^2-test $P: 0.20$, RMSEA: 0.03, CFI: 0.99, SRMR: 0.03.

[c] See supplemental material, Figure S3 and S5. Goodness-of-fit (same effect model): χ^2-test $P: 0.60$, RMSEA: ≤ 0.01, CFI: 1.00, SRMR: 0.02.

[d] See supplemental material, Figure S4 and S5. Goodness-of-fit (same effect model): χ^2-test $P: 0.32$, RMSEA: 0.02, CFI: 0.99, SRMR: 0.03
